# NeuroFlex: Feasibility of EEG-Based Motor Imagery Control of a Soft Glove for Hand Rehabilitation

**DOI:** 10.3390/s25030610

**Published:** 2025-01-21

**Authors:** Soroush Zare, Sameh I. Beaber, Ye Sun

**Affiliations:** 1 Department of Mechanical and Aerospace Engineering, University of Virginia, Charlottesville, VA 22903, USA; cyj7tf@virginia.edu (S.Z.); azq6wr@virginia.edu (S.I.B.); 2 Department of Electrical and Computer Engineering, University of Virginia, Charlottesville, VA 22903, USA

**Keywords:** brain–machine interface, EEG, motor imagery, deep learning, soft actuator, rehabilitation

## Abstract

Motor impairments resulting from neurological disorders, such as strokes or spinal cord injuries, often impair hand and finger mobility, restricting a person’s ability to grasp and perform fine motor tasks. Brain plasticity refers to the inherent capability of the central nervous system to functionally and structurally reorganize itself in response to stimulation, which underpins rehabilitation from brain injuries or strokes. Linking voluntary cortical activity with corresponding motor execution has been identified as effective in promoting adaptive plasticity. This study introduces NeuroFlex, a motion-intent-controlled soft robotic glove for hand rehabilitation. NeuroFlex utilizes a transformer-based deep learning (DL) architecture to decode motion intent from motor imagery (MI) EEG data and translate it into control inputs for the assistive glove. The glove’s soft, lightweight, and flexible design enables users to perform rehabilitation exercises involving fist formation and grasping movements, aligning with natural hand functions for fine motor practices. The results show that the accuracy of decoding the intent of fingers making a fist from MI EEG can reach up to 85.3%, with an average AUC of 0.88. NeuroFlex demonstrates the feasibility of detecting and assisting the patient’s attempted movements using pure thinking through a non-intrusive brain–computer interface (BCI). This EEG-based soft glove aims to enhance the effectiveness and user experience of rehabilitation protocols, providing the possibility of extending therapeutic opportunities outside clinical settings.

## 1. Introduction

Neurological disorders such as strokes and spinal cord injuries affect millions of people around the world, leading to severe motor impairments that limit hand and finger mobility, significantly affecting daily activities and quality of life [1]. Neuroplasticity, also known as brain plasticity, is the intrinsic capability of the nervous system that enables the brain to adjust to new experiences and compensate for lost functions [2]. Thus, integrating voluntary cortical activity, motor execution, and motion-related feedback is an effective approach to guide motor relearning for rehabilitation [3], which can be achieved by aligning device responses with the patient’s brain activity, potentially driving targeted adaptations in the motor cortex [4].

Robot-assisted rehabilitation has proven effective for stroke rehabilitation [5,6,7]. Conventional rehabilitation techniques and devices tend to be inflexible and cumbersome, reducing user adherence and constraining long-term rehabilitation efficacy [8,9]. Soft gloves that replicate the hand’s natural movement have garnered significant attention in recent years as a solution for effective and comfortable rehabilitation [10,11,12]. These flexible devices provide a valuable way for hand rehabilitation and fine motor practice while overcoming the discomfort of rigid systems [12,13]. Recently, the design and control of such soft gloves have experienced significant growth, aiming to support different recovery stages [14]. These soft designs enable customizable rehabilitation processes, demonstrating the feasibility of personalized assistive technology development [15,16,17].

Robotic devices for hand rehabilitation have demonstrated their effectiveness due to their potential to improve hand motor capabilities through repetitive exercises [18,19,20,21]. A number of wearable soft gloves have been successfully developed in recent years, offering enhanced safety, adaptability to hand sizes, usability in personal settings, and reduced cost. These gloves have adopted different actuation methods, ranging from cable/fiber-driven, elastomer-based, to pneumatic actuators. Among them, pneumatic designs have unique advantages, including easy fabrication, compressibility, ease of storage, lightweight properties, and an environmentally benign nature, all of which facilitate rapid actuation [22]. With these characteristics, soft pneumatic gloves provide the necessary flexibility and adaptability for effective hand rehabilitation exercises while maintaining safety and portability [23,24,25].

There are various methods used to activate assistive devices in rehabilitation, considering the patient’s capabilities in different rehabilitation stages. Gu et al. developed a hand exoskeleton rehabilitation system activated by a one-touch button [26]. Mohammadi et al. developed an intraoral tongue–computer interface (ITCI) for eye-free control of a five-degree-of-freedom (DOF) upper-limb exoskeleton [27]. Additionally, biosignals such as surface electromyography (sEMG) provide a way to detect user motor intent by measuring electrical muscle activity, allowing users to control exoskeletons [28]. Sunny et al. developed an eye-gaze control system for a 6-DOF wheelchair-mounted assistive robot, enabling individuals with motor impairment to perform activities of daily living [29].

These activation methods have provided accessible and practical human inputs for controlling assistive devices. To fully engage neural processes in motor execution, which are crucial for restoring the movements needed in daily tasks, there has been growing interest in coupling brain–computer interfaces (BCIs) with assistive robot control [4,30]. BCI-based approaches provide a more direct connection to the brain’s motor regions, aligning assistive device control with the neural mechanisms underlying motor function and offering the potential to enhance rehabilitation efficiency [31,32,33].

Among the various BCIs, electroencephalography (EEG) is a non-invasive method that measures biopotentials generated by the synchronous firing of large groups of neurons, allowing for the capture of motor intentions [34,35]. EEG-based BCIs provide real-time feedback, aligning the rehabilitation process with the patient’s motor intent by continuously monitoring neural signals from the motor cortex. Also, it has been shown that the repeated practice of MI activates sensorimotor regions similar to actual task performance and promotes neuroplasticity in the brain [36]. EEG-driven MI training can enhance neuroplasticity in motor pathways and improve hand function recovery in stroke patients by strengthening dorsal pathway connectivity [37].

The complexity and high dimensionality of EEG signals make them challenging to interpret, and the accuracy of task-specific classification is often not high. Methods such as linear discriminant analysis (LDA) [38], support vector machines (SVMs) [39], and common spatial patterns (CSPs) [40] rely on manual feature extraction and may show inconsistency in accuracy across subjects. Advanced deep learning techniques address these challenges by automatically extracting complex features from raw EEG data. Additionally, these models learn to adapt to individual neural patterns associated with MI tasks, achieving more consistent classification accuracy across subjects.

Deep learning models, including Convolutional Neural Networks (CNNs) and hybrid architectures, are effective for decoding MI from raw EEG data by automatically extracting both spatial and temporal features, which are essential for real-time applications [41,42,43]. Zhang et al. proposed a hybrid deep neural network model using transfer learning to address subject variability, achieving high classification accuracy with minimal training data by using a CNN and Long Short-Term Memory (LSTM) [44]. Khademi et al. implemented a CNN-LSTM model that uses transfer learning and continuous wavelet transforms for robust feature extraction and classification, even with limited data [45]. These models are advantageous in rehabilitation applications, where individual thought-process differences lead to distinct EEG patterns across subjects. Their ability to generalize and adapt to these variations makes deep learning a valuable tool for advancing EEG-based rehabilitation systems. CNNs typically require large datasets, so many studies use data augmentation, while transfer learning provides an alternative solution to adapt models effectively with limited data.

Transformer-based architectures have recently gained popularity due to their ability to capture long-range dependencies in sequential data [46,47]. Transformers use self-attention mechanisms to capture relationships across the entire sequence rather than focusing on localized features, making them particularly well suited for decoding MI tasks. Unlike CNNs, which extract local spatial features but may struggle to capture global temporal dependencies, transformers analyze the entire sequence, capturing both spatial and temporal features effectively. Similarly, LSTMs process sequences step by step, whereas transformers can analyze entire sequences in parallel, significantly improving computational efficiency. Moreover, temporal information is preserved by the use of positional encoding in the transformers, which is essential for time-sensitive data like EEG.

In this study, we develop a motion-intent-controlled soft glove, namely NeuroFlex, that is capable of detecting and assisting patients’ attempted movements using MI EEG. In order to overcome class imbalance difficulties, NeuroFlex uses a transformer-based model with focal loss, which ensures robust performance even with limited and unbalanced data. The EEG-based system directly engages the brain’s motor cortex, supporting a more intuitive and natural recovery process by aligning device control with the user’s motor intent. The transformer-based architecture further improves the accuracy of decoding MI from EEG signals, effectively modeling long-range dependencies within the data for a more precise response.

## 2. Methodology

### 2.1. EEG Data Acquisition and Experimental Protocol

**Figure 1:**
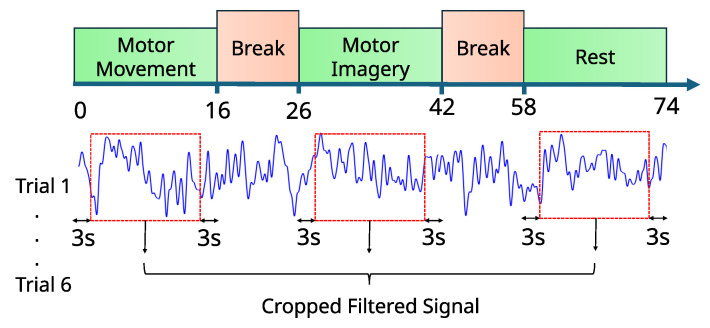
The experimental protocol for EEG data collection consisted of three phases: motor execution, motor imagery, and rest, each lasting 16 s.

The EEG data for this study were collected using a dry-contact EEG cap equipped with 16 ThinkPulse active EEG electrodes (OpenBCI Inc., Brooklyn, NY, USA). The electrode placement followed the international 10–20 system [48]. A Cython Daisy board was used to collect data from the 16 channels, and data acquisition was carried out using Python via the BrainFlow library to interface with the hardware and streamline the data collection process. Three healthy adult subjects participated in this study in order to validate feasibility. All subjects were made to feel comfortable throughout the data collection process to reduce the possibility of fatigue-induced interference with EEG signals. Inclusion criteria ensured that participants had no history of neurological disorders and were in good health during the experiment. Informed consent was collected before participation, and this study was approved by the Institutional Review Board (IRB) of the University of Virginia under protocol number 7070. Participants were instructed to engage in an MI hand rehabilitation task, which consisted of three phases. The experimental protocol is shown in Figure 1, which involved distinct phases: motor movement, MI, and rest. These tasks, randomized to ensure variability and robustness, were designed to capture EEG signals related to both physical and imagined glove movements, with cropped segments to remove initiation artifacts and enhance signal clarity.

In the first phase, participants were asked to physically perform a glove-closing and opening movement. This movement was performed at their own comfortable pace and frequency to simulate a rehabilitation task. This phase lasted 16 s, during which each participant conducted the movement naturally. In the second phase, participants were instructed to mentally imagine the same movement of closing and opening their gloves, without executing any physical action, for 16 s. During the final phase, i.e., the rest phase, participants were asked to relax completely and avoid any physical or mental activity. This allowed the EEG signals to return to their baseline state. This phase lasted for 16 s.

For each phase, the data’s first and last 3 s were removed to eliminate transitional effects caused by the participants initiating or completing the tasks. Each subject completed six trials of the aforementioned protocol, with each trial including all three phases. The process was repeated in three separate sessions, resulting in a total of 18 trials per subject.

**Figure 2:**
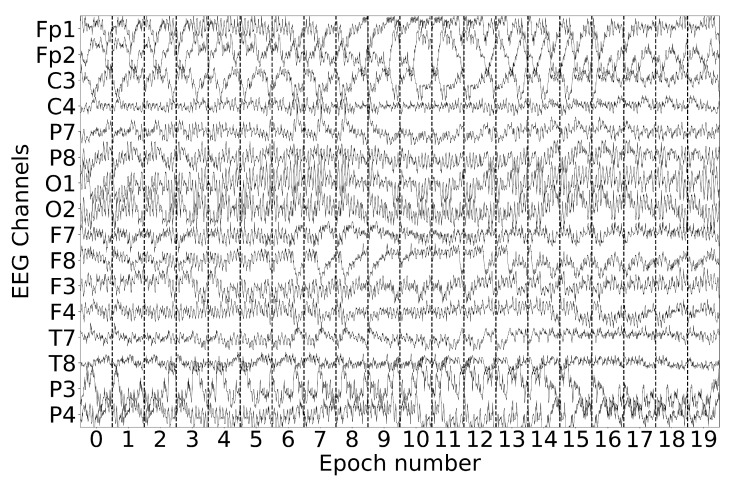
Raw EEG data during glove MI after bandpass filtering (0.5–45 Hz) and notch filtering (60 Hz), segmented into overlapping 1-second epochs with a 0.75-second overlap.

Figure 2 shows the raw EEG data recorded during an MI task where the subject was asked to think about performing a glove movement. The EEG signal was preprocessed with a bandpass filter ranging from 0.5 to 45 Hz to retain the relevant neural frequency bands associated with MI. In addition, a 60 Hz notch filter was applied to remove interference from the power line. To maintain temporal continuity and make cross-epoch analysis easier, the data were segmented into 1-second overlapping epochs with a 0.75-s overlap.

### 2.2. Transformer-Based Architecture

A transformer-based deep learning model was used to capture motion-related activity from all 16 EEG channels. This approach processed all 16 channels of EEG data rather than concentrating on specific brain regions, which can vary significantly between individuals and across applications. The transformer-based architecture in this study was designed to handle EEG data’s unique temporal and frequency characteristics for classifying MI tasks. The core of the architecture uses the self-attention mechanism, allowing it to model long-range dependencies in time-series data for decoding preprocessed EEG signals.

The architecture began by transforming the raw EEG signals into the frequency domain using the Fast Fourier Transform (FFT). This step allowed the model to focus on specific neural frequency bands. The frequency bands used for MI tasks were delta (0.5–4 Hz), theta (4–8 Hz), alpha (8–13 Hz), beta (13–30 Hz), and gamma (30–45 Hz), which are known to correspond to different cognitive and motor functions [49,50].

The 1–45 Hz frequency band was selected as the optimal range for MI decoding through a trial-and-error process. This decision was driven by the observation that narrowing the frequency range toward higher or lower bands led to a reduction in classification accuracy. Including low-frequency bands (delta and theta) and high-frequency bands (beta and gamma) provided a more comprehensive view of the neural activity during MI and rest phases. The rest phase and MI task were better differentiated when the entire 1–45 Hz band was considered, potentially because narrowing the focus to specific bands may exclude valuable information across multiple frequencies for different subjects.

Positional encoding is incorporated to add temporal information to the model. Positional encoding ensures that the model retains the sequence order of the EEG data, which is required to understand the nature of the EEG time series for that specific epoch. The encoding is applied as an additive term to the input embeddings, allowing the model to differentiate between different time steps.

At the core of the model are multiple transformer encoder blocks. Each block consists of two main components:

- Multi-Head Attention: This layer allows the model to focus on different parts of the EEG sequence simultaneously, helping it capture complex temporal patterns. The attention mechanism works by projecting the input into query, key, and value matrices and then computing a weighted sum of these values. With its multi-head design, the attention layer can analyze multiple parts of the sequence concurrently, enhancing the model’s ability to capture different signal aspects.
- Feed-Forward Neural Network (FFNN): After the attention layer, the output passes through a feed-forward network consisting of two dense layers with a non-linear activation function. These layers enable the model to capture more complex data features. Dropout and layer normalization are applied to prevent overfitting.

After the encoder blocks, a global average pooling layer is applied to reduce output dimensionality. Global average pooling takes the averages across the temporal dimension, efficiently narrowing the learned features. These pooled features then pass through dense layers before the final classification.

A focal loss function is employed during training to address the class imbalance in the EEG dataset. Focal loss mitigates the issue of class imbalance by giving more weight to complicated EEG signals that are hard to classify, ensuring that minority classes, such as the MI task, are well-represented and used in the training process. Combining Fast Fourier Transform (FFT) preprocessing, positional encoding, multi-head attention, and focal loss enhances the effectiveness and accuracy of the model in interpreting EEG signals.

**Figure 3:**
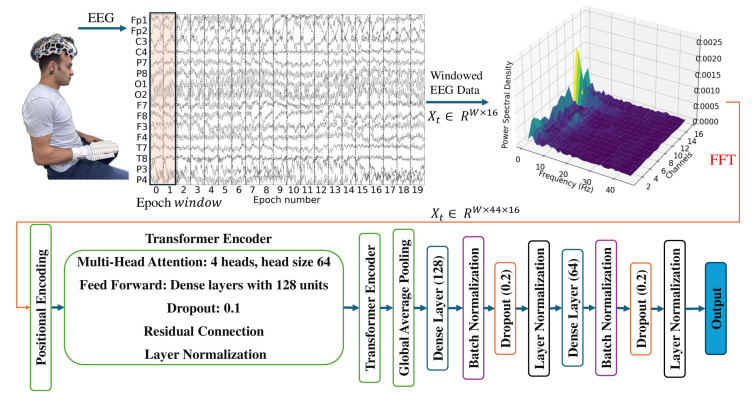
Overview of EEG signal processing using a transformer architecture.

Figure 3 illustrates the transformer-based architecture for processing EEG signals, incorporating the FFT to filter the EEG data. The architecture comprises a multi-head attention mechanism with four heads, each with a size of 64, followed by dense layers containing 128 units and dropout layers to prevent overfitting. A transformer encoder with positional encoding processes the sequential EEG data. Subsequent layers, including batch normalization, dense layers, and residual connections, aim to improve the model’s learning efficiency and stability. The output layer represents the final predictions based on the processed EEG signals, facilitating tasks like motor imagery classification.

The architecture is summarized in Table 1. The input layer is a matrix of shape (44, 16), where the first dimension represents the 44 frequency bins derived from the FFT of the raw EEG data, capturing information across different frequency ranges from 1 to 45 Hz. The second dimension corresponds to the 16 EEG channels. These frequency bins are required for capturing distinct patterns associated with MI, as different neural frequency bands (delta, theta, alpha, beta, and gamma) reflect different cognitive and motor processes. By transforming the time-domain EEG signals into frequency bins, the architecture is able to exploit both spatial and temporal features of the data, allowing the self-attention mechanism to attend to key frequency components over time and enhancing the accuracy of MI decoding. Each subsequent layer, including the multi-head attention, dropout, and normalization layers, processes this frequency input.

The primary advantage of the transformer architecture over CNNs is its ability to capture long-range dependencies in sequential data. Unlike CNNs, which rely on localized convolutions, transformers use attention mechanisms to focus on different parts of the input sequence, making them more flexible and powerful for time-series data. Furthermore, transformers can generalize well across different datasets and participants, making them more robust for real-world rehabilitation scenarios where EEG data may vary.

## 3. Design and Fabrication of the Soft Glove

The design and fabrication of the soft actuating system focus on developing a soft robotic glove for performing rehabilitation tasks. In this section, three primary processes are discussed: the design of the soft fingers and the glove base, the manufacturing technique used, and the assembly of the glove base with the five fingers to produce the complete soft pneumatic glove. Additionally, to ensure that the overall system is robust, flexible, and performs the same functions as the human hand, 3D fused deposition modeling (FDM) printing parameters were optimized, along with the optimized geometry and materials for soft printing.

### 3.1. Design of the Soft Fingers

Different designs and structures can be used to represent pneumatic soft fingers [51,52]. In this work, we chose the pneumatic networks (PneuNets) structure due to its flexibility and compliance with rehabilitation functions using its internal inflatable chambers and channels. The design and control of PneuNets are crucial for providing the required motion that adapts to the motion of human fingers during rehabilitation or the grasping process when well-pressurized. The soft, flexible material that has been used for the manufacturing of the soft fingers was the commercial thermoplastic poly(urethane) TPU 85A (NinjaTek, Manheim, PA, USA), which is commonly used for such 3D printing processes [51].

**Figure 4:**
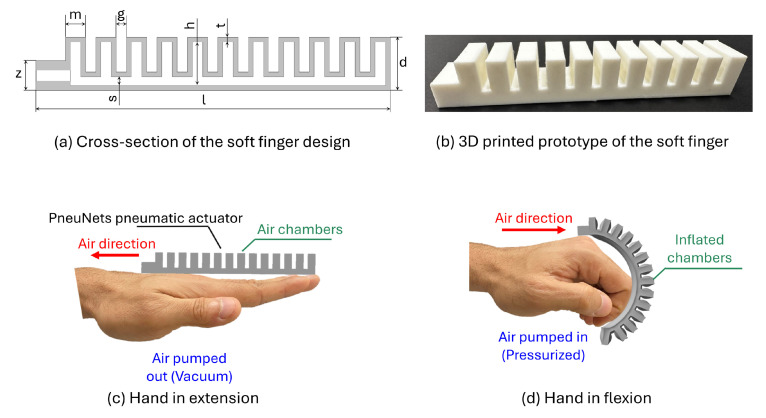
Design and conceptual function of the soft fingers in this study.

In this study, the model of the pneumatic soft fingers was carefully designed using the 3D CAD computer-aided engineering (CAE) software (SOLIDWORKS 2022). The detailed optimized design of the soft fingers, which captures the essential features necessary for accurate functionality, is illustrated in Figure 4a. Additionally, the specific and optimized parameters chosen for this design are thoroughly detailed in Table 2. The parameters that describe the design of the soft fingers are the finger length (l), finger height (d), air channel height (s), chamber thickness (m) and height (h), and the gap between the chambers (g). The lengths of the five soft fingers are as follows: 99 mm for the thumb, 140 mm for the index and ring fingers, 151 mm for the middle finger, and 120 mm for the little finger. The thickness of the wall sides is the same as that of the chamber wall (t). Figure 4b shows the 3D-printed prototype of one of the soft pneumatic fingers, which can be used immediately after printing without postprocessing. The conceptual function of the soft glove, as shown in Figure 4c,d, involves using a PneuNets pneumatic actuator with segmented air chambers to mimic finger joints, facilitating bending and extension for hand rehabilitation. When pressurized, the actuator drives the fingers into flexion, and when vacuumed, it aids in returning the fingers to their extended positions.

### 3.2. Three-Dimensional Printing Process and Parameters

Soft robot fabrication structures, especially soft actuators, have been created using various techniques, such as shape deposition manufacturing (SDM) [53] and molding [54,55]. The main material used in SDM and molding is silicon rubber due to its flexibility and safety. However, the fabrication process using SDM and molding can be difficult and time-consuming if the structure of the soft robot is complex. Also, to achieve the required shape or design, precise design guidelines are still required. On the other hand, additive manufacturing (AM) or 3D printing represents a promising fabrication method for any complex geometry and can handle soft robot structures [56,57]. Three-dimensional printing techniques are widely used nowadays with various shapes, including selective laser sintering (SLS), powder bed fusion (PBF), stereolithography (SLA), and fused deposition modeling (FDM).

The low-cost 3D printing process was performed using an open-source FDM printer (PRUSA Inc., Prague, Czech Republic). The approach to achieving an airtight soft finger can be summarized in two steps: calibrating the first layer and optimizing the printing settings. Each soft finger was printed on its side without the need for support material and was ready for use without postprocessing. The optimized printing parameters for the PRUSA Slicer (version 2.8.1) are shown in Table 3.

### 3.3. Design of the Full Glove

**Figure 5:**
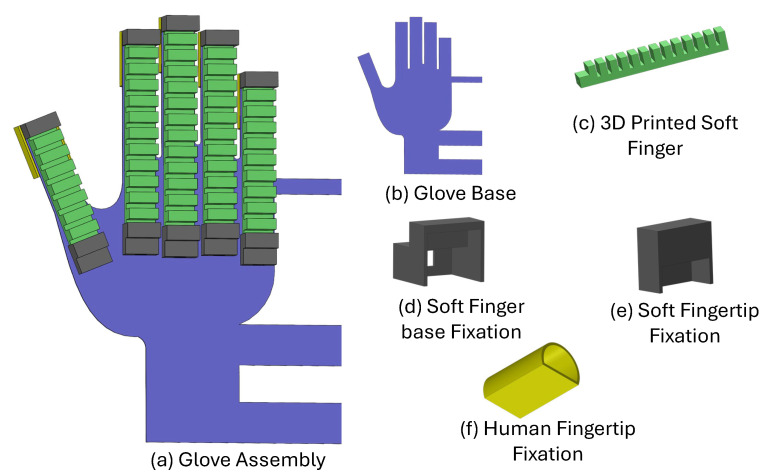
Assembly of the full glove from the CAD software, including all the parts used for the actuation process.

The complete actuating system, as described by the full glove design, was achieved by integrating the five soft fingers with the 3D-printed glove base to mimic human hand flexibility and natural movements. The main parts of the design are shown in Figure 5. One key component is the base, which provides structural support and controls the system’s movements. The base design accommodates five fingers with dimensions suitable for different hand sizes, and each finger base is 19 mm wide to match the human hand or glove profile. Moreover, the base, soft fingertip, base fixation, and human finger fixation were printed as one part to ensure both flexibility and strength.

The soft fingertip and base fixation serve to securely hold the soft fingers in place during operation and to control and limit finger extension during deformation. The human fingertip fixation provides the interaction point for the human fingers with the soft glove. This design allows the hand to grip objects in a controlled and safe manner, ensuring that even patients with limited strength can perform effective rehabilitation exercises without straining muscles or causing tissue damage. The total weight of the assembled glove is approximately 240 g, making it lightweight and portable. All the parts of the soft glove are made from commercial thermoplastic poly(urethane) TPU 85A (NinjaTek, Manheim, PA, USA).

**Figure 6:**
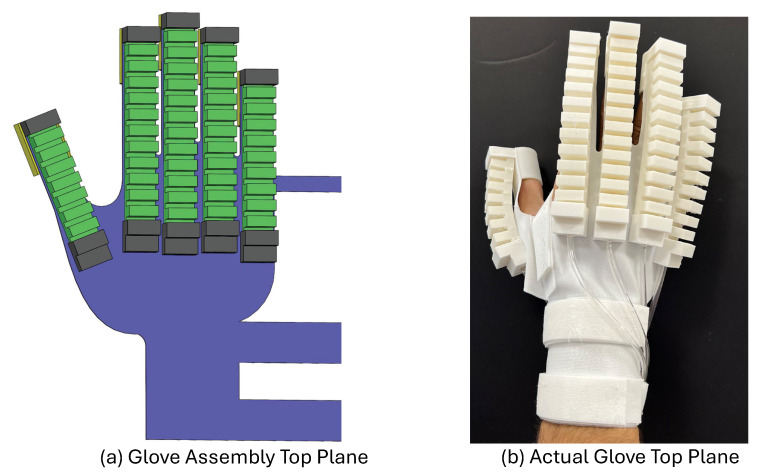
Comparison of the top plane of the full glove for the (**a**) glove assembly CAD model and (**b**) the actual glove design.

**Figure 7:**
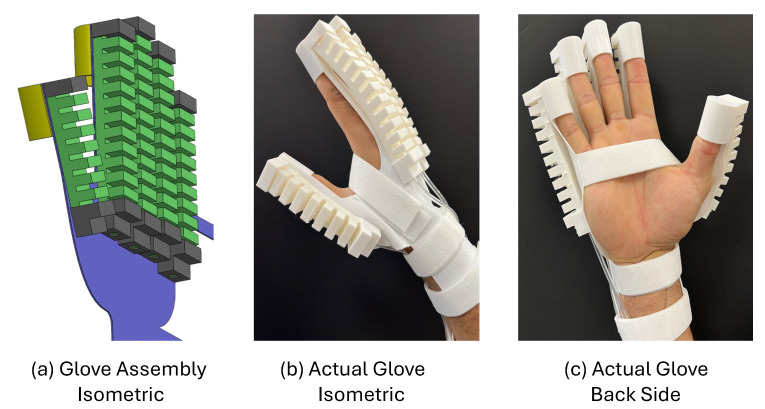
Different views and comparison of the full glove: (**a**) Isometric view of the CAD model. (**b**) Isometric view of the actual design. (**c**) Back view of the actual side.

To visualize the functionality, a comparison between the CAD model and the actual printed prototype is shown in Figure 6 and Figure 7. Figure 6 compares the CAD assembly of the full glove with the actual printed prototype, showing how the fingers are fixed and placed within the design space to maintain flexibility and ensure full functionality for actuation and rehabilitation functions. Also, Figure 7 shows a more detailed view of the complete glove design, comparing the isometric view of the CAD model with the real prototype and showing how the human fingers integrate with the prototype safely, firmly, and securely. Overall, these comparisons highlight the transition from the CAD model to a working prototype, demonstrating the system’s ability to perform rehabilitation tasks with fully controlled and precise mechanisms. This design guarantees the ability to perform various functions, including grasping, releasing, and customized motion patterns.

## 4. Rehabilitation Control Loop

EEG technology plays a vital role in health assistance applications by allowing the system to react in real time to the patient’s intentions and re-establishing the brain’s ability to control motor functions, which is a critical aspect of recovery without physical exertion. The hand rehabilitation control process uses EEG signals initialized with signal acquisition through an EEG cap with 16 channels placed on the patient’s head. The aim of the EEG cap is to detect brain activity related to the process of thinking about starting the rehabilitation process by opening and closing the glove or reaching rest mode and then to convert these signals into electrical inputs that initiate the rehabilitation process and control the overall rehabilitation process.

The classification results of the EEG data, derived from the transformer-based deep learning model, are used to determine the participant’s motor intent. These classification results are translated into control signals that activate the soft robotic glove’s pneumatic actuation system. When the trained transformer deep learning model predicts a “fist” intent, the computer sends a signal via a microcontroller (Arduino Uno) connected to the pneumatic pump system. This signal activates the pressurized pump, inflating the glove’s soft actuators to close the fingers. Similarly, when the model predicts a “rest” state, the computer sends a signal to activate the vacuum pump, which deflates the actuators, allowing the glove to return to its neutral state. The data transmission between the computer and the pump system is through USB communication with the microcontroller. The microcontroller sends the control signals to the pneumatic valves to ensure smooth and timely operation of the glove. This closed-loop system enables real-time, reliable, and precise control of the glove’s movements, providing effective rehabilitation aligned with the participant’s intent to increase neuroplasticity.

**Figure 8:**
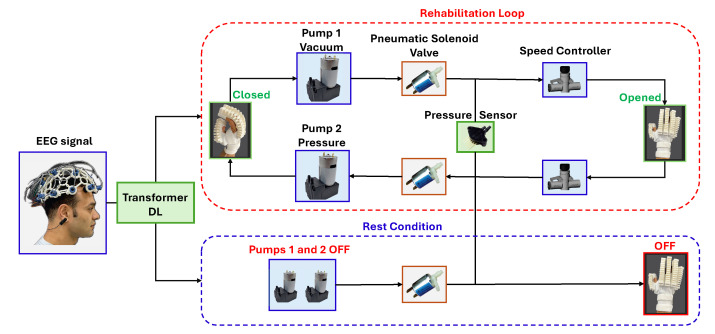
Schematic diagram and optimized control process for the rehabilitation loop and the rest conditions.

Controlling this process involves analyzing and filtering out the noise from the EEG signals to ensure accurate interpretation and then converting the signals into commands through the transformer DL model for the control system, as shown in Figure 8. The rehabilitation loop, which is the first and primary step in this process, is initiated upon receiving the motion signal from the brain by activating two pneumatic pumps: one for pressurizing to assist with closing the hand and the other for creating a vacuum to return the hand to its original shape. For the second condition, which is the rest condition, the two pumps are turned off, and the air is vented from the system to allow the hand to return to an open position, simulating natural extension and release motions, ensuring that the soft glove moves only when intended. The soft pneumatic actuators used in the soft glove design are optimized to react smoothly to these signals, creating gentle and safe hand movements, which are critical for effective rehabilitation without overexertion or risk of injury. The angle of the glove’s deformation can be gradually adjusted by increasing the pressure step by step or gradually setting a maximum pressure, assisting in good recovery. The gradual increase in the deformation angle follows approaches used by Park et al. [58] and Becker et al. [59].

The optimized system consists of two pneumatic pumps: one for pressurizing to assist with closing the hand and the other for creating a vacuum to return the hand to its original shape. Two pneumatic solenoid valves (CJV23-C12A1, Conjoin, Xiamen, China) are connected with the pumps to control the airflow throughout the system, and a third one is for venting the air under rest or safety conditions. A flow speed pneumatic valve (AS2002F-04A, SMC Corporation, Noblesville, IN, USA) is connected to each pump to control the speed of the air and keep the motion smooth and safe for the patient. A pressure sensor (MPX5500DP, NXP, Austin, TX, USA) is integrated to accurately control the system. This sensor continuously monitors the pressure within the system, ensuring it stays within a predefined range. If the pressure exceeds a maximum predefined amount or drops below a minimum amount, the initial pressure sensor reading, the sensor triggers the system to vent and stop, preventing injury. Maintaining the correct pressure is critical to ensure the deformation of the soft actuators remains within safe limits suitable for rehabilitation. The system uses these thresholds to maintain the optimal level of deformation, providing effective hand movement while protecting the patient from over-compression or excessive vacuum, both of which could cause harm. The pneumatic flow control valves and the pressure sensor work together to ensure smooth, controlled, and safe motions, enhancing the rehabilitation process’s effectiveness and safety, as described in Algorithm 1.
**Algorithm 1** Real-time monitoring and pressure control for pneumatic actuators1:Initialize Pressure Sensor $P_{s}$, Pneumatic System State $P_{0}$2:Set Pressure Limits $P_{\max}=$ Pre-defined value, $P_{\min}=$ Initial reading3:Get EEG Signal Processing Model: $S_{\theta }$4:**while** patient in rehabilitation session **do**5:    $s_{t}\leftarrow S_{\theta }\left(e_{t}\right)$                                  ▹ Process EEG Signal6:    **if** $s_{t}=1$ **then**                              ▹ Patient intends to close hand7:        **if** $P_{t}<P_{\max}$ and $P_{t}>P_{\min}$ **then**8:            Activate Pneumatic Pump 1                     ▹ Pressurize to close hand9:            Increase pressure: $P_{t}=P_{t}+\Delta P$10:        **else if** $P_{t}\geq P_{\max}$ **then**11:            Activate Pneumatic Pump 2                        ▹ Vacuum to open hand12:            Decrease pressure: $P_{t}=P_{t}-\Delta P$13:        **end if**14:    **else if** $s_{t}=0$ **then**                             ▹ Patient intends to rest15:         Maintain or reset pressure as needed                    ▹ No active command16:    **end if**17:    **Safety Check:**18:    **if** $P_{t}>P_{\max}$ **then**19:         Trigger safety valve to release excess pressure20:         Stop system and vent air21:    **else if** $P_{t}<P_{\min}$ **then**22:         Activate vacuum vent to stabilize pressure23:    **end if**24:**end while**

## 5. Results

**Figure 9:**
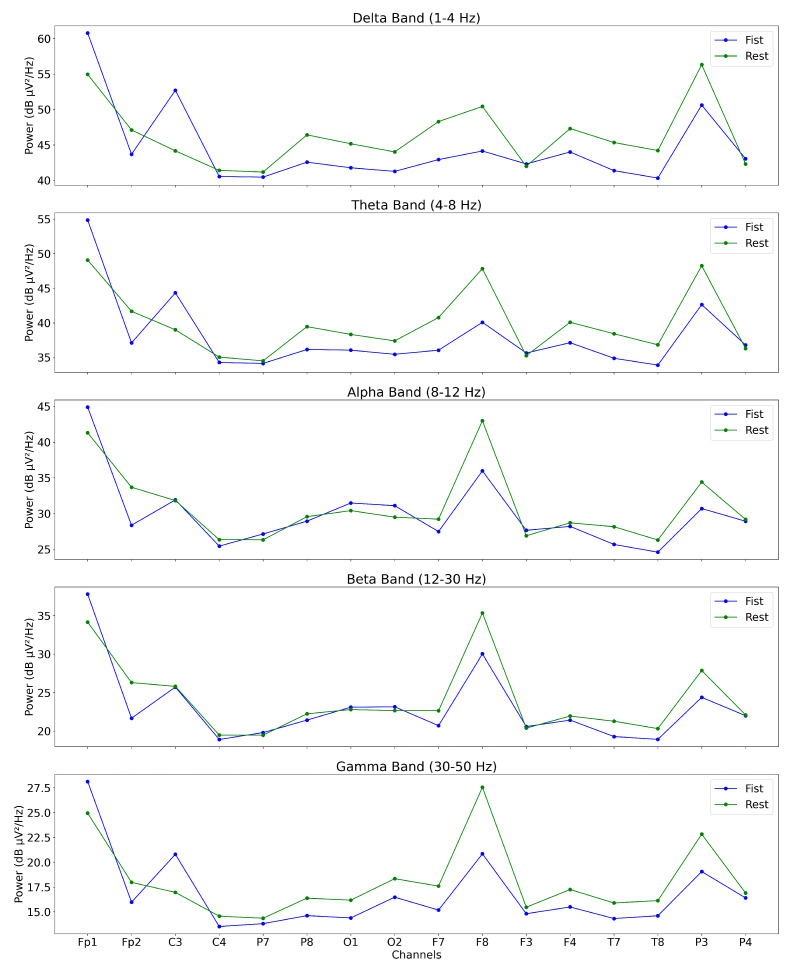
EEG band power for Subject ID 1 across frequency bands.

Figure 9 illustrates the band power distribution of EEG data for Subject ID 1, comparing the power across specific frequency bands (delta, theta, alpha, beta, and gamma) during “glove” MI and rest conditions. Only MI data were analyzed, with any actual glove movement data excluded to capture neural patterns associated solely with the imagined action. The delta and theta bands display more precise distinctions between conditions, with noticeably higher power levels during rest conditions in these lower frequency ranges. Furthermore, channels F7, F8, and F3 exhibit more significant differentiation in the alpha, beta, and gamma bands between MI and rest conditions. This suggests that these channels may be pivotal in distinguishing MI from rest states. This analysis emphasizes the importance of frequency-specific brain activity and strategic channel selection in EEG studies focused on MI.

The performance metrics are presented in Table 4, providing insights into the model’s effectiveness for MI classification across subjects by assessing the F1 score, Cohen’s Kappa, accuracy, and Area Under the Curve (AUC). For Subject ID 1, the model achieved the highest accuracy, with an F1 score of 0.8513, Cohen’s Kappa of 0.7065, accuracy of 0.8533, and AUC of 0.9449. Comparatively, Subject ID 2 had lower accuracy across all metrics, possibly due to a lack of concentration during the experiment, the subject’s mood, or a complicated pattern of the brain and variability in EEG signal patterns for the same motor intention. Subject ID 3’s results were intermediate. This variability shows the potential benefits of personalized model adjustments to maximize performance across individuals. Also, individually trained models are needed since different people think differently about the same motor intentions.

The variation in F1 scores and Cohen’s Kappa values between subjects suggests individual variability in EEG patterns, which could be attributed to physiological or cognitive differences in MI. For instance, Subject ID 2 showed a relatively lower Cohen’s Kappa (0.5164), which may indicate more variance in prediction consistency. This result implies that, while the model performed well, further fine-tuning of the model could enhance its consistency and reliability and make it practical for use in real-time applications that require reliable and accurate interpretations of motor intent. Table 5 provides a comparison of NeuroFlex with other works, highlighting its applicability and the advantages of its transformer-based architecture compared to traditional methods, such as CNN, LSTM, GRU, and CSP, in decoding MI from EEG data.

**Figure 10:**
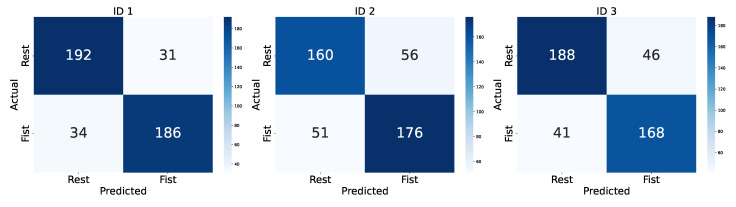
Confusion matrices for participants.

Figure 10 shows the confusion matrices for each subject, representing the model’s ability to predict “fist” and “rest” states accurately. For ID 1, the model classified 192 out of 223 rest epochs correctly, whereas for ID 2, rest classification accuracy dropped slightly. The model’s misclassification rates were relatively low across the subjects, which shows the robust capability of the model to differentiate MI tasks effectively.

**Figure 11:**
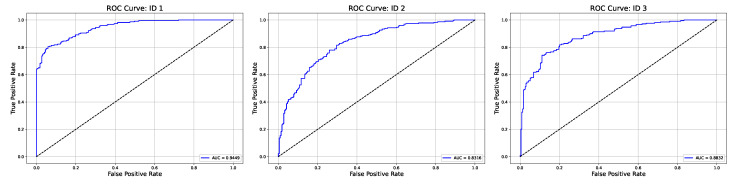
ROC curves for participants.

Figure 11 presents the ROC curves for each ID, demonstrating the model’s ability to differentiate between MI classes across various threshold settings. The ROC curves are near the upper-left corner across all subjects. These curves show the model’s consistency in identifying MI patterns with high sensitivity, which is essential for reliable and practical EEG-based control systems in rehabilitation applications. Combining the ROC analysis with the confusion matrices provides a comprehensive view of the model’s performance and effectiveness across MI tasks and subjects.

## 6. Conclusions and Discussion

This paper presents NeuroFlex, an EEG-based MI control system that controls a soft robotic glove for hand rehabilitation. NeuroFlex uses EEG data to interpret motor intent, converting them into real-time control commands for a wearable exoskeleton. This enables patients to perform rehabilitation exercises, allowing them to engage in therapeutic processes without physical exertion. The system’s core utilizes a transformer-based model designed to decode EEG signals. With its self-attention mechanism, this architecture is suitable for capturing long-range dependencies in sequential data, effectively distinguishing MI patterns. FFT preprocessing is incorporated to focus on frequency bands associated with motor tasks, which improves the model’s accuracy in identifying MI tasks.

Performance metrics across multiple subjects reveal NeuroFlex’s robustness and adaptability. The model achieves high values in the F1 score, Cohen’s Kappa, accuracy, and AUC, indicating reliable performance in classifying MI and rest conditions across individuals. Confusion matrices and ROC curves further validate its capacity for accurate distinction, supporting their applicability in real-time rehabilitation. Future directions may include individualized model calibration to further optimize NeuroFlex’s performance. This study highlights NeuroFlex’s potential as a non-invasive, adaptable, and efficient tool for supporting patients in hand rehabilitation following neurological impairments.

## Figures and Tables

**Table 1:** Transformer-based architecture used for MI classification.

Layer (Type)	Output Shape	Param #	Connected to
Input Layer	(None, 44, 16)	0	-
Add	(1, 44, 16)	0	Input Layer
Multi-Head Attention	(1, 44, 16)	17,168	Add
Dropout 1	(1, 44, 16)	0	Multi-Head Attention
Add 1	(1, 44, 16)	0	Dropout 1, Add
Layer Normalization	(1, 44, 16)	32	Add 1
Dense	(1, 44, 128)	2176	Layer Normalization
Dropout 2	(1, 44, 128)	0	Dense
Dense 1	(1, 44, 16)	2064	Dropout 2
Add 2	(1, 44, 16)	0	Dense 1, Layer Normalization
Layer Normalization 2	(1, 44, 16)	32	Add 2
Multi-Head Attention 2	(1, 44, 16)	17,168	Layer Normalization 2
Dropout 3	(1, 44, 16)	0	Multi-Head Attention 2
Add 3	(1, 44, 16)	0	Dropout 3, Layer Normalization 2
Global Avg. Pooling	(1, 16)	0	Add 3
Dense 2	(1, 128)	2176	Global Avg. Pooling
Batch Normalization	(1, 128)	512	Dense 2
Dropout 4	(1, 128)	0	Batch Normalization
Layer Normalization 3	(1, 128)	256	Dropout 4
Dense 3	(1, 64)	8256	Layer Normalization 3
Batch Normalization 2	(1, 64)	256	Dense 3
Dropout 5	(1, 64)	0	Batch Normalization 2
Layer Normalization 4	(1, 64)	128	Dropout 5
Dense 4	(1, 2)	130	Layer Normalization 4

**Table 2:** Geometry of the soft fingers.

z	m	g	h	t	d	s
10 mm	6.8 mm	3.5 mm	13.8 mm	1.6 mm	17 mm	2 mm

**Table 3:** Three-dimensional printing parameters for the PRUSA Slicer, version 2.8.1.

Parameter	Value	Unit
Print settings		
Layers and perimeters		
Layer height	0.1	mm
First layer height	0.2	mm
Vertical shells perimeters	4	-
Horizontal shells top	16	-
Horizontal shells bottom	15	-
Extra perimeters on overhangs	Enabled	-
Avoid crossing perimeters	Enabled	-
Thick bridges	Enabled	-
Infill		
Fill density	100	%
Enable ironing	Enabled	-
Ironing type	All top surfaces	-
Flow rate	15	%
Speed		
Speed for print moves	30	%
Speed for non-print moves	120	%
First layer speed	20	%
Advanced		
Infill/perimeters overlap	10	%
Filaments		
Filament		
Temperature		
First and other layers	240	°C
Bed	50	°C
Cooling		
Bridges’ fan speed	90	%
Filament overrides		
Retraction length	2.5	mm
Retraction speed	60	mm/s
Deretraction speed	25	mm/s
Minimum travel after retraction	3	mm
Printers		
Nozzle diameter	0.4	mm

**Table 4:** Performance metrics for participants.

ID	F1 Score	Cohen’s Kappa	Accuracy	AUC
1	0.8513	0.7065	0.8533	0.9449
2	0.7669	0.5164	0.7585	0.8316
3	0.7943	0.6065	0.8036	0.8832

**Table 5:** Comparison of NeuroFlex with other works.

Work	Accuracy	Number of Actions and Tasks	Method
NeuroFlex	0.8051	2 (Fist, Rest)	Transformer DL (this method)
Ang et al. [60]	0.6800	4 (Left hand, Right hand, Feet, Tongue)	Filter bank CSP
Schirrmeister et al. [61]	0.7200	4 (Left hand, Right hand, Feet, Tongue)	CNN with cropped training
Amin et al. [62]	0.7380	4 (Left hand, Right hand, Feet, Tongue)	CCNN
Lu et al. [63]	0.7662	4 (Left hand, Right hand, Feet, Tongue)	CNN and LSTM
Qiao and Bi [64]	0.7662	4 (Left hand, Right hand, Feet, Tongue)	Bidirectional GRU
Liao et al. [65]	0.7460	4 (Left hand, Right hand, Feet, Tongue)	Shallow CNN

## Data Availability

The original contributions presented in this study are included in the article. For further information, including access to the code and the glove model, please visit the associated GitHub repository at https://github.com/SoroushZare/NeuroFlex (accessed on 1 January 2025).
